# Significance of RAS Mutations in Thyroid Benign Nodules and Non-Medullary Thyroid Cancer

**DOI:** 10.3390/cancers13153785

**Published:** 2021-07-27

**Authors:** Vincenzo Marotta, Maurizio Bifulco, Mario Vitale

**Affiliations:** 1 UOC Clinica Endocrinologica e Diabetologica, AOU S. Giovanni di Dio e Ruggi D’Aragona, 84131 Salerno, Italy; vincenzo.marotta@sangiovannieruggi.it; 2 Department of Molecular Medicine and Medical Biotechnology, University of Naples Federico II, 80100 Naples, Italy; maubiful@unina.it; 3 Department of Medicine, Surgery and Dentistry, University of Salerno, 84081 Baronissi, Italy

**Keywords:** RAS, FNAC, thyroid cancer, nodular goiter, indeterminate cytology

## Abstract

**Simple Summary:**

Only about 4% of thyroid nodules are carcinomas and require surgery. Fine-needle aspiration cytology is the most accurate tool to distinguish benign from malignant thyroid nodules, however it yields an indeterminate result in about 30% of the cases, posing diagnostic and prognostic dilemmas. Testing for genetic mutations, including those of RAS, has been proposed for indeterminate cytology to solve these dilemmas and support the clinician decision making process. A passionate debate is ongoing on the biological and clinical significance of RAS mutations, calling into question the utility of RAS as tumor marker. Recently, the description of a new entity of non-invasive follicular thyroid neoplasm and the accurate review of more recent analyses demonstrate that RAS mutations have limited utility in both the diagnostic and prognostic setting of thyroid nodular disease.

**Abstract:**

Thyroid nodules are detected in up to 60% of people by ultrasound examination. Most of them are benign nodules requiring only follow up, while about 4% are carcinomas and require surgery. Malignant nodules can be diagnosed by the fine-needle aspiration cytology (FNAC), which however yields an indeterminate result in about 30% of the cases. Testing for RAS mutations has been proposed to refine indeterminate cytology. However, the new entity of non-invasive follicular thyroid neoplasm, considered as having a benign evolution and frequently carrying RAS mutations, is expected to lower the specificity of this mutation. The aggressive behavior of thyroid cancer with RAS mutations, initially reported, has been overturned by the recent finding of the cooperative role of TERT mutations. Although some animal models support the carcinogenic role of RAS mutations in the thyroid, evidence that adenomas harboring these mutations evolve in carcinomas is lacking. Their poor specificity and sensitivity make the clinical impact of RAS mutations on the management of thyroid nodules with indeterminate cytology unsatisfactory. Evidence suggests that RAS mutation-positive benign nodules demand a conservative treatment. To have a clinical impact, RAS mutations in thyroid malignancies need not to be considered alone but rather together with other genetic abnormalities in a more general context.

## 1. Introduction

The majority of thyroid nodules are benign and require only clinical and ultrasonographic follow up [1]. In few cases it is a cancer whose clinical behavior can be extremely indolent or sometime aggressive. Fine needle-aspiration cytology (FNAC) is the gold standard for diagnosis [2]. Unfortunately, 5–10% of FNAC are not diagnostic because of inadequate sampling and 20% to 25% do not clearly indicate the nature of the lesion, so that these nodules are classified as indeterminate, posing difficulties for management [3,4].

Testing for genetic mutations has been proposed for indeterminate cytology to improve FNAC accuracy and for prognostic purpose, supporting the clinicians’ decision making process. Mutations of rat sarcoma viral oncogene homolog (RAS), rearranged during transfection/PTC (RET/PTC), v-Raf murine sarcoma viral oncogene homolog B1 (BRAF), telomerase reverse transcriptase (TERT), and rearrangement of paired box gene 8-peroxisome/proliferator-activator receptor γ (Pax8/PPARγ), are some of the genetic mutations useful to improve the diagnostic accuracy of needle biopsy when added to cytological evaluation. For this reason, mutational analysis is recommended in the last revisions of the guidelines for the diagnosis and management of thyroid nodules with indeterminate cytology [5,6]. The most popular molecular test used is the seven-gene panel of mutations (including RAS, BRAF, RET/PTC, and Pax8/PPARγ) with an estimated sensitivity of 57–75%, specificity of 97–100%, PPV of 87–100%, and NPV of 79–86% [7,8,9].

Although RAS mutations are included in these tests, they have low diagnostic sensitivity and specificity by themselves, benign nodules harboring RAS mutations have uncertain malignant potential, and the role of mutated RAS in the clinical outcomes of thyroid cancer is uncertain. This scenario has been further complicated by the recent introduction of a new histology category of encapsulated follicular variant of papillary thyroid carcinoma, with highly indolent behavior. For all these reasons, the significance of RAS mutations and how to appropriately use them to assist the management of thyroid nodules and thyroid cancer is unclear.

The purpose of this review is to present a comprehensive view of the vast and often discordant literature on RAS mutations in thyroid nodular disease. Specifically, we will discuss the oncogenic role of RAS mutations, their clinical significance, and potential utility as a diagnostic and prognostic tool in the management of benign nodules and non-medullary thyroid cancer.

## 2. Oncogenicity of Ras Mutants

Ras proteins are small GTPases which comprise three Ras proto-oncogenes: Harvey, Kirsten, and Neuroblastoma rat sarcoma viral oncogene homologs (HRas, NRas, and KRas). Ras is a guanine nucleotide transferase involved in different intracellular signaling pathways including the mitogen-activated protein kinase (MAPK) pathway and lipid kinases. Through these pathways, Ras regulates several crucial physiological processes including cell growth, differentiation, apoptotic cell death, oxidative stress, DNA damage response, adhesion, cytoskeletal rearrangements and cell motility [10,11]. 

RAS mutants have been found in 95% of pancreatic ductal adenocarcinomas, 50% of colon cancers, and 30% of non–small cell lung cancers (NSCLC). Overall, activating mutations in RAS are found in 32% of human cancers (https://cancer.sanger.ac.uk/cosmic, accessed on 9 June 2021). 

**Figure 1:**
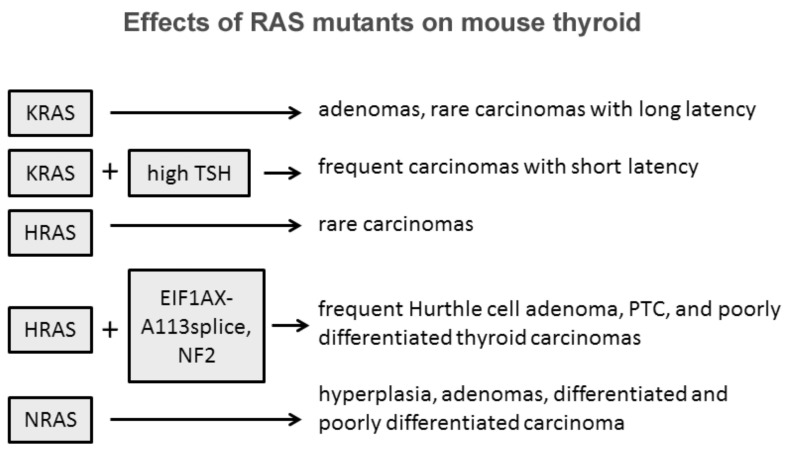
Experimental models of mice injected with retrovirus carrying mutant RAS, or transgenic mice expressing mutant RAS, alone or treated with goitrogenic factors increasing the THS or expressing additional factors.

The role of Ras in thyroid cell differentiation and tumorigenesis has been demonstrated in animal experimental models (Figure 1). Only 10% percent of rats whose thyroid was injected with a retrovirus carrying mutant KRAS developed thyroid carcinomas of very small size and with a one-year latency. Only the treatment of the animals with a goitrogenic agent induced thyroid carcinomas within three months in 90% of the animals. Transgenic mice bearing mutated KRAS under the control of rat thyroglobulin promoter developed only thyroid adenomas at low frequency and long latency, while the frequency increased with a concomitant treatment with a goitrogenic agent as occurred in retrovirus-injected animals [12]. These results demonstrate a low oncogenic potential of KRas mutants in the thyroid, boosted by a cofactor as high TSH stimulation. Similar results with the development of rare papillary thyroid carcinomas were obtained with mutant HRas under the control of bovine thyroglobulin promoter [13]. These results suggest that HRas and KRas mutations alone are not sufficient to induce thyroid carcinomas, while they could act as a predisposing factor. According to this hypothesis is the observation that isolated expression of HRasG12V or loss of NF2 are insufficient to drive thyroid tumorigenesis, while their coexpression in the thyroid induces large thyroid cancers, mostly poorly differentiated, with high penetrance [14]. One more example of cooperation among RAS mutants and other effectors was proposed by Krishnamoorthy et al. [15]. Mice expressing the eukaryotic initiation factor 1A hotspot splice-site mutation (EIF1AX-A113splice) developed only thyrocytes hyperplasia with atypical features, whereas mice coexpressing EIF1AX-A113splice and HRasG12V developed Hurthle cell adenomas, papillary thyroid carcinomas (PTC), and poorly differentiated thyroid cancer (PDTC) with high penetrance and close phenocopy of human thyroid tumors harboring the combined genetic lesions [16].

Some evidences suggest that among the three isoforms, NRAS mutations are those more oncogenic in the thyroid. Transgenic mice expressing mutated human NRAS^G61^ specifically in thyroid follicular cells developed progressive lesions from follicular cell hyperplasia to adenoma and carcinoma [17]. Thirty percent of mice developed differentiated tumors with follicular or mixed papillary-follicular malignant features and 10% of mice developed invasive carcinomas with large poorly differentiated areas. Furthermore, distant metastases were observed in the liver of three mice, in the lung of two mice, and in the femur of one mouse. This striking difference between RAS isoforms might be explained with the intrinsic tissue-specific different isoform effects and role of members of the RAS family as suggested by transgenic mice experimental models where RAS expression was not tissue-restricted [18,19,20,21]. 

Overall, these experimental models demonstrate that RAS overexpression and RAS mutants represent a favorable genetic ground in thyroid follicular cells and that the collaboration of other effectors and/or genetic events is necessary in conferring the cancer phenotype. Among RAS isoforms, NRAS displays a stronger albeit limited oncogenic potential, a conclusion supported also by studies in spontaneous thyroid tumors.

## 3. Prevalence of RAS Mutations in Thyroid Nodular Disease

Thyroid cancer is among the first tumors where activating RAS mutations were recognized [22,23]. Quite soon it appeared clear that RAS mutations were detectable at all stages of thyroid tumorigenesis, being found in 33% of adenomas, 53% of differentiated follicular carcinomas and 60% of undifferentiated carcinomas [24]. 

Although there is a general agreement that RAS mutations are present at any stage of thyroid oncogenesis, the range of prevalence is quite broad as a consequence of pathology classification and the sensitivity of detection method applied. In early studies, polymerase chain reaction-single-strand conformational polymorphism (SSCP), in many cases followed by DNA sequencing, was the most frequently used method for the detection of RAS mutations. This method yielded a lower prevalence with respect to sequencing in all histotypes but FTC, revealing the presence of an RAS mutation in about 2.3% of nodular hyperplasia (NH) (1/43), 13% of follicular adenomas (FAD) (13/103), 10% of PTC (16/165) and 26% of follicular thyroid carcinomas (FTC) (11/43) [25,26,27,28,29,30,31]. Direct sequencing of PCR products resulted in a higher percentage of RAS mutations: 9.3% in NH (11/115), 22% in FAD (56/257), 23% in PTC (115/508) and 29% in FTC (36/125) [32,33,34,35,36,37,38,39,40,41]. 

Many studies on surgical samples with a definitive diagnosis have been performed to assess the prevalence of all RAS mutations in benign and malignant thyroid nodules (Table 1). In 2296 tissue samples, NRAS is the isoform most frequently mutated (59.4% of all RAS mutations), and among all activating point mutations NRAS61 is the most frequent (56.9%). The prevalence of RAS mutation in tissue samples of NH, FAD, FTC, and PTC were, respectively, 8.5%, 21.0%, 29.8%, and 11.5%.

In the context of differentiated thyroid cancer (DTC), representing almost the totality of follicular-cell derived thyroid malignancies [49], the detection of RAS mutations is related to the follicular architecture. Indeed, mutated RAS is not only present in a relevant portion of FTC, but among PTCs, it is more frequently detected in the follicular variant (FVPTC) [46,50], characterized by a nearly total follicular pattern coupled with cytological PTC features. Actually, RAS mutations are typically present in the encapsulated FVPTC (EFVPTC), whereas the unencapsulated/infiltrative FVPTC (IFVPTC) frequently carry the BRAFV600E mutation and are characterized by more aggressive behavior [51,52]. 

Recently, the malignant nature of EFVPTC came into question. A large panel of experienced pathologists classified it as a separate entity, the non-invasive follicular thyroid neoplasm with papillary-like nuclear features (NIFTP) exhibiting a very indolent and perhaps non-malignant nature [53,54,55]. After a 13 year follow-up, no recurrence occurred in 109 patients with NIFTP, none of them were treated with radioactive iodine ablation and only a minority with total thyroidectomy, while 12 of 101 patients with invasive EFVPTC had adverse follow-up events in 3.5 years. Following these observation, the 2017 WHO Classification of Endocrine Organ Tumours included NIFTP in the group of the so-called “follicular-patterned neoplasms with borderline clinical behavior”, assigning to this neoplasm a very low malignant potential, with minimal risk of recurrence or metastasis [56]. This generated great interest among researchers dealing with thyroid cancer, trying to define genetics of such new entity. 

Based on available evidence from several studies of molecular profiling, a relevant portion of NIFTPs carry RAS mutations. In fact, the review of 18 studies [52,53,57,58,59,60,61,62,63,64,65,66,67,68,69,70,71,72] showed a wide range of prevalence for RAS mutations, from 20 to 100%. This could be related to the heterogeneity of tumor series coming from different geographic areas and different source (cytology samples or tumor specimens), and detection method (direct sequencing, PCR-based approaches, or next-generation sequencing [NGS] technology). However, out of a total of 488 NIFTPs, RAS mutations were detected in 242 cases, a percentage as high as 49.6%. 

## 4. Role of RAS Mutations in the Diagnosis of Indeterminate Thyroid Nodules

The tiered Bethesda classification scheme has been widely adopted among pathologists to yield a standardized interpretation of FNAC for thyroid nodules [73]. However, three of six diagnostic categories do not lead to a definite characterization: atypia of uncertain significance/follicular lesion of undetermined significance (AUS/FLUS, Class III), follicular neoplasm/suspicious for follicular neoplasm (FN, Class IV), and suspicious for malignant cells (SMC, Class V) [4,73,74,75,76]. Indeed, while false-negative rates for benign cytology (Class II) and false-positive rates for malignant cytology (Class VI) are very low (<5%), cancer risks for such indeterminate categories range greatly, engendering a dilemma, difficult to solve in the clinical practice. 

Testing for point mutations, translocations in genomic DNA or gene expression profiling using RNA from thyroid nodules have been extensively investigated in the attempt to refine inconclusive cytology. 

However, because of cases selection, the ratio between tumor histotypes and methodological issues, sensitivity and specificity of RAS detection tests are variable among the studies. 

Sensitivity is particularly affected by the detection method applied and by the ratio between cancers with follicular architecture (including FTC and FVPTC) and classic PTC histotype. Sensitivity was very low in two studies (5.8% and 11.8%) [35,77]. In one of them [35], this could rely on a methodological issue, as DNA from tissue samples was analyzed by direct sequencing of PCR amplicons, an approach limited by the possibility of false negative results in case of low tumor cells fraction. However, in both series, the FTC histotype was poorly represented (FTC/PTC = 8/46 and 3/7, respectively), a reason for which the prevalence of RAS mutations was low, thus affecting sensitivity. Conversely, a sensitivity as high as 48.4% was obtained in the Nikiforov et al. study, which used real-time LightCycler PCR for analysis of 93 malignant nodules, where the tumors with a follicular architecture were the majority (78/93) [7]. Additionally, the specificity displays a large variability among the studies, ranging from 27.3 to 97.8% [7,78,79]. The high specificity of the Nikiforov report [7], achieving 97.8%, can be explained by the low ratio between FAD and hyperplastic nodules (HN) (the latter harboring RAS mutations rarely) among the benign nodules analyzed (FAD/HN, 107/261). Similarly, the high ratio FAD/HN (22/15) can explain the lower specificity (78.4%) obtained in the study by Gill et al. [79]. 

The positive predictive value (PPV) is the percentage of patients with a positive test result who actually have the disease. Conversely, the negative predictive value (NPV) is the percentage of patients with a negative test who actually do not have the disease. In the clinical setting, PPV is the best way for the clinician to rule in a disease while NPV is the best way to rule it out. The PPV and NPV of tests that employ the detection of RAS mutants in indeterminate nodules is quite variable, ranging from 92.3 to 42.9% and from 87.9 to 61.7%, respectively. Again, such large differences are explained by ratio between tumor histotypes, methodological issues and, also, sample numerosity. 

**Figure 2:**
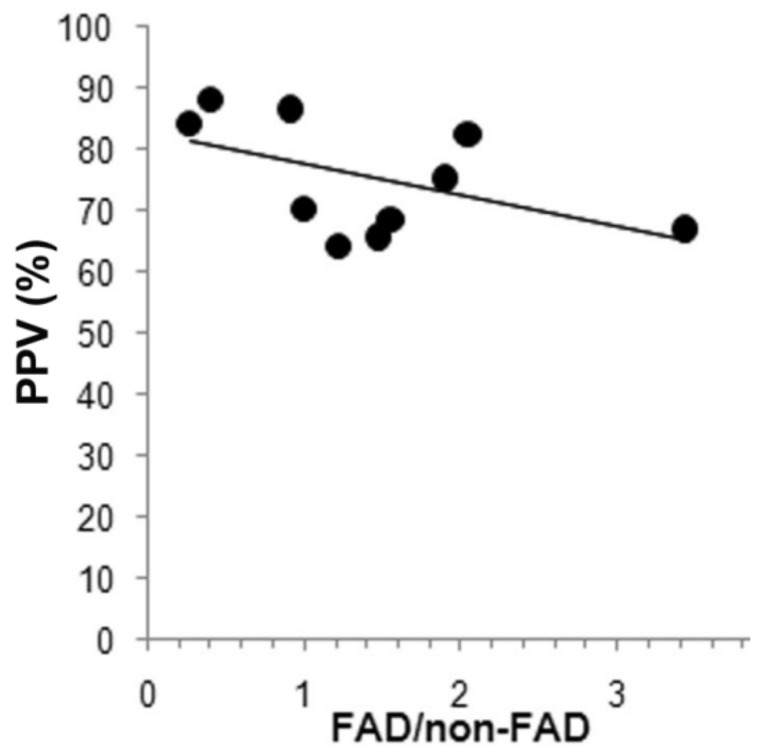
Relationship between positive predictive value (PPV) and the ratio follicular adenoma/non-follicular adenoma benign nodules (FAD/benign non-FAD). R2 = 0.088, *p* = 0.032.

Among the studies with higher sample numerosity, Eszlinger applied the seven-gene panel in a series of 247 class III and IV indeterminate nodules [80]. In this study, RAS mutations were present in 8.5% of nodules, a percentage comparable with that of Cantara and Nikiforov (7.3% and 11.5%, respectively) [7,81], and almost double that reported by Liu et al. (4.5%) [35]. In this series, despite the high prevalence of malignant tumors with a follicular architecture (74/84), which should empower sensibility, NPV was only 68.6%. By contrast, despite FAD being abundant among benign nodules (99/163), therefore compromising specificity, PPV was 61.9%. Consistently, the cumulative analysis of 17 studies including 1966 indeterminate FNAC and corresponding histology demonstrates the following performance: sensitivity 37.9%, specificity 91.0%, PPV 77.8%, NPV 75.3% (Table 2). Overall, the sensitivity of RAS mutations is low in all studies (12.5–66.7%), thus the test is not able to rule out a thyroid cancer and avoid inappropriate surgery. By contrast, its specificity is robust enough to reinforce the risk of cancer, even though PPV is extremely variable depending on the composition of benign nodules in the series analyzed. The higher the ratio FAD/benign non-FAD nodules is, generally including HN and Hashimoto thyroiditis, the lower PPV is as a consequence of the high prevalence of RAS mutations in FAD and low prevalence in benign non-FAD nodules. Hence, high PPV is reached in the series where benign non-FAD nodules are abundant, the nodules that most frequently yield a class II Bethesda cytologyy but that some time can yield indeterminate cytology. The FAD/benign non-FAD ratio of 11 studies including 1062 nodules is negatively correlated to PPV as shown in Figure 2. Thus, the PPV and the NPV and the possibility to rule out and rule in a cancer by a test based on the detection of RAS mutations depends on the composition of the nodules with indeterminate cytology and ultimately depends on the experience of the pathologist that analyzed and assigned the cytology class.

In such a context, an opened question is whether and how the introduction of the NIFTPs can impact on the significance of mutated RAS as predictor of malignancy in indeterminate nodules. Whether RAS mutations are more frequent in NIFTP than in invasive EFVPTC is still uncertain. Out of 11 studies addressing this issue, a higher frequency was reported in five papers [52,61,67,71], whereas no difference or a lower prevalence was reported in seven [59,60,62,63,65,68,70]. However, the shift of NIFTPs from the malignant to non-malignant thyroid tumors is expected to lower the specificity of mutated RAS, thereby impairing its main strength point as diagnostic test. Such speculation has been confirmed by at least two studies [65,92]. In 2013, when the NIFTP histology had not been introduced yet, Gupta et al. [92] searched for RAS mutations by means of real-time Lightcycler PCR on a wide prospective series of consecutive cytology specimens. Out of 61 RAS-mutated patients, histology revealed malignancy in 50 cases, with an impressive PPV of 82%. Of note, the vast majority of samples carrying RAS mutations yielded indeterminate cytology (Bethesda classes III, IV, and V [4]) (63/68 aspirates), so such diagnostic performance may be referred to the inconclusive FNAC category. Among the 50 malignant cases, a number as high as 44 were diagnosed with FVPTC (44/50), and 29 cases were described as non-invasive encapsulated, which is consistent with the current definition of NIFTP. When removing such patients from the malignant nodules, PPV dramatically dropped to 34.5%. The 2017 study by Valderrabano et al. [65] reported molecular analysis by means of ThyroSeq v2 (a NGS approach which simultaneously tests for point mutations in 13 genes, including RAS, and for 42 types of gene fusions) on 102 cytology specimens with indeterminate result (Bethesda classes III and IV [4]), subsequently resected. Since tumors with follicular architecture were dominant (NIFTP-FVPTC-FTC/other histotypes, 13/20), RAS mutations were the most frequent in this series (47%). Comparative analysis of the scenarios including or not NIFTPs within the malignant nodules showed a significant reduction in PPV, falling from 31.2 to 25%. Similarly, PPV of the whole ThyroSeq v2 analysis dropped from 42 to 33% if considering NIFTP as benign. 

Although mutated Ras does not enable a diagnosis of malignancy, its presence increases the risk of malignancy of indeterminate nodules, thus suggesting a more strict surveillance or surgery, according to clinical evaluation. 

Combinational use of RAS mutations with other genetic markers is a strategy proposed for the diagnosis of thyroid cancer. A mutation testing using a seven-gene panel, including RAS, did not improve the presurgical diagnosis of thyroid indeterminate FNAC, also because of the low percentage of RAS mutation-positive carcinomas and high percentage of RAS mutation-positive benign nodules [80]. The previously mentioned ThyroSeq v2 was tested on 143 class IV indeterminate nodules [93]. The performances of the test were high: PPV 83% and NPV 96%. However, the contribution of RAS was minor because of its low sensitivity (51.3%).

## 5. Role of RAS Mutations in the Prognosis of Benign and Malignant Thyroid Nodules

The higher prevalence of RAS mutations in thyroid carcinomas with respect to adenomas, and the experimental evidence of the tumorigenicity of RAS mutations in transgenic mice, albeit weak, tissue selective, and RAS isoform dependent, raise a reasonable concern about the prognosis of cytologically benign or indeterminate thyroid nodules harboring RAS mutations. The convincement that mutant RAS may drive progression from follicular adenoma to carcinoma and to further tumor dedifferentiation is supported by the finding that both PTC and FTC with poorly differentiated areas which showed higher rates of RAS mutations [44,46]. Furthermore, it was found that mutated RAS determines chromosome instability [94], and this may explain its possible role in promoting dedifferentiation. Owing to these evidences, the surgical removal of the adenomas carrying RAS mutations would be justified for preventing tumor progression. However, direct evidence that RAS mutation-positive adenomas evolve in carcinomas is lacking to date. Very few studies on the evolution of RAS-positive benign nodules can be found in the literature. In one of these, 24 RAS mutation-positive and 54 RAS mutation-negative thyroid nodules with benign cytology were followed for a mean of 25-months (range 13–47 months) [95]. RAS mutation-positive nodules displayed faster growth (*p* < 0.001), but no signs of tumor progression were seen. Most of those patients are still under clinical and ultrasonographic surveillance and, after a mean follow-up of about 6 years, none displayed signs of progression (unpublished data). Nine histologically or cytologically benign RAS mutation-positive thyroid nodules were followed for a mean duration of 8.3 years (range 3.1–24.0 years) [96]. All displayed indolent behavior with no significant growth, or ultrasonographic suspicious features including abnormal adenopathy. A second cytology was performed at mean 5.8 years later in four nodules, confirming no significant change in cellular morphology. 

Although the number of studies on the evolution of benign nodules harboring RAS mutations is still very limited for patient numerosity and follow-up duration, the stability observed so far challenges the hypothesis that RAS mutation is a risk factor for malignant transformation. The natural history of cytologically benign thyroid nodules with unknown mutational status argues against the role of RAS mutations as a risk factor for thyroid cancer. In a retrospective study on 330 cytologically benign nodules followed for a mean of 20 months (range 1 month–5 years), many of them re-evaluated by a second FNAC, only one was demonstrated to be a PDTC [97]. One hundred benign solid nodules did not display signs of risk for thyroid cancer over a 9 to 11 years follow-up and of 21 nodules re-examined by FNAC only one case turned out to be malignant [98]. In a prospective study involving 992 consecutive patients with cytologically benign thyroid nodules of unknown RAS mutational status, only five nodules proved to be malignant after 5 years of follow-up, that is, only 0.3% of all nodules [99]. The spare reclassified cases are compatible with a misdiagnosis and are much lower than the number of RAS mutation-positive nodules expected, suggesting that RAS mutation is not a risk factor for the malignant evolution of benign nodules. To date, in the absence of evidence that cytologically benign nodules bearing RAS mutations evolve toward malignancy or have a different progression, testing the RAS mutational status is not recommended. A limit of these studies is that the majority of them have been performed in nodules with benign cytology and these conclusions cannot be automatically extended to nodules with indeterminate cytology, where other genetic mutations can coexist with RAS mutations.

Prognostic studies of RAS mutational status in thyroid cancer generated conflicting results. Earlier studies have reported the association between RAS mutations and a more aggressive phenotype of thyroid cancer [100]. In a series of 88 carcinomas, an NRAS mutation was present in 13.9% of well-differentiated carcinomas (WDTC), in 18.2% of PDTC and 37.5% of undifferentiated carcinomas [101]. In the study by Garcia-Rostan et al. a RAS mutation was found in 8.2% of WDTC, in 55.2% PDTC, and in 51.7% of undifferentiated or anaplastic carcinomas (UTC) [45]. A cumulative analysis of 834 thyroid cancers including 492 WDTC, 173 PDTC and 169 UTC thyroid cancers, displayed a RAS mutation in 151 tumors (Table 3). RAS mutations were more frequent in PDTC (27.7%) then in WDTC (14.8%) and UTC (17.7%), confirming the hypothesis that RAS is involved in thyroid cancer progression or it is simply associated with it, whereas the undifferentiated/anaplastic thyroid carcinoma does not necessarily arise from a WDTC and it is more linked to other genetic alterations. However, the dilemma whether de-differentiation of WDTC is accompanied by RAS mutations or RAS promotes the de-differentiation in spontaneous thyroid cancer is unsolved.

Some studies have reported the association between RAS mutations and more aggressive behavior of thyroid cancer and a higher frequency of distant metastases. Five (33%) of 15 patients who died of PTC exhibited an RAS mutation, whereas this mutation was present in only eight (10.5%) of the 76 patients still alive after a 35 year follow-up [47]. In a 2012 study focused on 58 FTC, Fukahori et al. [107] found RAS mutations associated with distant metastases and poor overall survival. More recently, a study of 56 FTC, half of which with distant metastases, found an independent relationship between mutations at codon 61 of NRAS and distant metastases [108]. However, a limit of all mentioned studies is the low numerosity of the samples. 

A large retrospective study including 1510 PTC followed for a mean of 33 months demonstrated less aggressive histological features, lower rates of extrathyroidal extension, and lower stage for RAS positive tumors, as compared to those with BRAF^V600E^ or RET/PTC [109]. However, TERT promoter mutations, which were recently demonstrated to be of strong prognostic significance [110,111,112,113], were not included in the analysis, and this represents a crucial limitation. The analysis of the impact of RAS mutation on clinicopathological outcome in 162 BRAF mutation-negative PTC patients unveiled that RAS mutation alone had no adverse effects [114,115]. The patients harboring an RAS mutation alone did not display a more frequent tumor recurrence or higher mortality rate when compared with patients with no RAS, BRAF or TERT promoter mutation, suggesting that RAS mutation alone is not associated with aggressive behavior. Notably, the coexistence of RAS mutations with TERT promoter mutations was strongly associated with late disease stages, distant metastasis, and recurrence, as compared with the group of PTC negative for either mutation. These data, in addition with the finding that RAS mutations is more frequent in NIFTP than in other differentiated thyroid carcinomas, support the notion that RAS mutation alone is not a risk factor for a worse prognosis, whereas its coexistence with additional mutations is associated with a poorer outcome.

## 6. Conclusions

Genetic studies and integrated genomic analysis have defined the landscape of molecular alterations in thyroid cancer. At the beginning, great enthusiasm was aroused by the discovery of driver oncogenes and by the possibility to use them to refine uncertain diagnosis and foresee the behavior of benign and malignant thyroid nodules. 

The physiological role of Ras as a component of the MAPK cascade and in other signaling in the thyroid cell made RAS mutations major actors in thyroid carcinogenesis. Experimental models in animals demonstrated a weak carcinogenetic potential of RAS mutations in the thyroid, and more importantly, their distribution in the entire spectrum of thyroid neoplasms from benign follicular adenomas to PDTC and anaplastic thyroid carcinomas made it clear fairly quickly that its utility as diagnostic marker is poor. Furthermore, the recent introduction of the NIFTP histotype has further weakened the accuracy of mutated RAS as a diagnostic tool. Despite this, RAS mutations are still considered in some diagnostic molecular tests. 

The significance of RAS mutations as a prognostic marker has changed in recent years. At the beginning, RAS mutations were considered a promoter of progressive dedifferentiation in benign nodules, and as such a risk factor for evolution from adenoma to carcinoma, therefore a marker of poorer prognosis in DTC. It is clear now that the impact of RAS mutations on thyroid cell, normal and transformed, and the impact on the clinical behavior of thyroid neoplasms must not to be considered by itself but rather together with other genetic abnormalities in a more complex contest. 

Comprehensive analysis on large series of thyroid carcinomas, as the one performed on PTC [114], is to date the more powerful tool to gain more insight into the role of RAS mutations in thyroid cancer development and its biological and clinical behavior. The characterization of minor and major genetic mutations, gene expression, and methylation that cooperate with RAS mutation will have diagnostic, prognostic and possibly therapeutic application.

## Figures and Tables

**Table 1:** RAS mutation studies in surgical samples that have been considered in the present review.

Study	Year	Method	Tumor Histotype	N	HRAS		KRAS		NRAS		All RAS	All RAS %
					12/13	61	12/13	61	12/13	61		
Lemoine, N.R. [24]	1989	SB	FAD	24	1	2	1	0	1	3	8	33.3
			FTC	10	1	2	0	0	0	1	4	40
Namba, H. [42]	1990	SB	PTC	14	3	0	0	0	0	0	3	21.4
			FTC	3	0	0	0	0	0	0	0	0
			NH	19	4	0	0	0	0	0	4	21.1
			FAD	24	4	0	0	0	0	2	6	25
Shi, Y. [43]	1991	SB	FTC	16	0	3	0	0	0	1	4	25
			PTC	22	0	0	0	0	0	0	0	0
			FAD	25	0	12	0	0	0	2	14	56
Yoshimoto, K. [28]	1992	SSCP	FAD	24	0	1	0	0	0	4	5	20.8
			PTC	26	0	0	0	0	0	0	0	0
Manenti, G. [36]	1994	SEQ	FTC	19	0	0	0	0	0	0	0	0
Ezzat, S. [25]	1996	SSCP	NH	18	0	0	0	0	0	0	0	0
			FAD	7	0	0	0	0	0	1	1	14.3
			PTC	16	1	0	0	0	0	1	2	12.5
			FTC	4	0	0	0	0	0	1	1	25
Naito, H. [29]	1998	SSCP	PTC	34	0	0	5	1	0	1	7	20.6
Bouras, M. [34]	1998	SEQ	FAD	35							0	22.9
			PTC	66							0	9.1
			FTC	6							0	16.7
Sugg, S.L. [26]	1999	SSCP	PTC	20	1					1	2	10
Esapa, C.T. [31]	1999	SSCP	NH	25	0	0	0	0	0	1	1	4
			FAD	41	0	0	0	2	0	5	7	18.4
			FTC	8	0	0	0	0	0	4	4	50
			PTC	12	0	0	0	0	0	1	1	8.3
			PTC	46	0					0	0	0
Motoi, N. [32]	2000	SEQ	FAD	5	0	0	0	0	0	2	2	40
			PTC	16	1	0	3	0	0	7	11	68.8
			FTC	4	0	0	0	0	0	1	1	25
Nikiforova, M.N. [44]	2003	FMCA	FAD	23		2				6	8	34.8
			FTC	33		2				12	14	42.4
			FTC	19		1	1				2	10.5
Vasko, V. [33]	2003	SEQ	FAD	46	0	0	0	0	0	7	7	15.2
			FTC	34	1	0	0	0	0	6	7	20.6
Soares, P. [27]	2003	SSCP	FAD	34							0	14.7
			PTC	27							0	7.4
			FTC	12							0	33.3
Garcia-Rostan, G. [45]	2003	SSCP	PTC	30							0	6.7
			FTC	19							0	10.5
Zhu, Z. [46]	2003	PCR	PTC FV	30	3					10	13	43.3
Liu, R.T. [35]	2004	SEQ	NH	20	0	0	0	0	0	0	0	0
			FAD	17	0	0	0	0	0	1	1	5.9
			PTC	42	0	0	0	0	0	0	0	0
			FTC	8	0	1	0	1	0	1	3	37.5
Fukahori, M. [47]	2012	LHSA	FAD	40	0	4	0	0	4	4	12	30
			FTC	58	2	3	3	2	1	22	33	56.9
Park, J.Y. [38]	2013	SEQ	PTC FV	132	0	4	0	8	1	22	35	26.5
Schulten, H.J. [40]	2013	SEQ	NH	18	0	1	0	0	0	0	1	5.6
			FAD	69	0	5	0	0	0	10	15	21.7
			PTC	89	0	4	0	1	0	7	12	13.5
			FTC	17	0	1	0	0	0	1	2	11.8
Giordano, T.J. [37]	2014	SEQ	NH	27							0	3.7
			FAD	28							0	17.9
			PTC	36							0	13.9
			FTC	17							0	17.6
McFadden, D.G. [39]	2014	SNS	PTC FV	127							0	36.2
Giordano, T.J. [37]	2014	NGS	PTC	403		14	2	1		34	51	12.9
Jeong, S.H. [41]	2018	PSEQ	NH	50	0	3				6	9	18
			FAD	57	0	5	5	1	1	10	22	31.6
			FTC	39	0	4	6	3	1	7	21	48.7
Duan, H. [48]	2019	NGS	FTC	10	1	2	0	0	0	1	4	40
			FAD	48		4				3	7	14.6
Lemoine, N.R. [24]	1989	SB	FAD	24	1	2	1	0	1	3	8	33.3
			FTC	10	1	2	0	0	0	1	4	40
Namba, H. [42]	1990	SB	PTC	14	3	0	0	0	0	0	3	21.4

NGS, Next-Generation Sequencing; PCR, Polymerase chain reaction; SSCP, Single-Strand Conformation Polymorphism; FMCA, LightCycler PCR and fluorescence melting curve analysis; SEQ, sequencing; SB, Southern blot; LHSA, PCR-based loop-hybrid mobility shift assay; PSEQ, pyrosequencing; SNS, SNaPshot multiplexed targeted sequencing. NH, nodular hyperplasia; FAD, follicular adenoma; FTC, follicular thyroid Cancer; PTC, papillary thyroid cancer.

**Table 2:** Analysis of RAS mutations in FNA samples of thyroid nodules with indeterminate cytology (class III and IV of Bethesda cytology).

Study	Year	N	% RAS Positive	RAS Pos Ben	RAS Pos Mal	RAS Neg Ben	RAS Neg Mal	Sensitivity	Specificity	Accuracy	PPV	NPV
Moses, W. [82]	2010	110	7.3	4	4	77	25	13.8	95.1	50.0	75.5	73.6
Cantara, S. [81]	2010	41	7.3	1	2	33	5	28.6	97.1	66.7	86.8	85.4
Nikiforov, Y.E. [7]	2011	461	11.5	8	45	360	48	48.4	97.8	84.9	88.2	87.9
Liu, S. ^a^ [77]	2014	50	10.0	4	1	38	7	12.5	90.5	20.0	84.4	78.0
Le Mercier	2014	32	18.8	2	4	24	2	66.7	92.3	66.7	92.3	87.5
Beaudenon-H., S. [9]	2014	41	24.4	2	8	22	9	47.1	91.7	73.2	80.0	71.0
Gill, M.S. [79]	2015	60	26.7	8	8	29	15	34.8	78.4	50.0	65.9	61.7
Stence, A.A. [83]	2015	20	15.0	1	2	12	5	28.6	92.3	66.7	70.6	70.0
De Napoli, L. [84]	2016	258	12.0	9	22	159	68	24.4	94.6	71.0	70.0	70.2
Shrestha, R.Y. [85]	2016	56	41.1	12	11	26	7	61.1	68.4	47.8	78.8	66.1
Eszlinger, M. [86]	2017	247	8.5	8	13	155	71	15.5	95.1	61.9	68.6	68.0
Patel, S.G. ^b^ [87]	2017	87	-	21	66	-	-	-	-	75.9	-	-
Valderrabano, P. [65]	2017	102	15.7	11	5	71	15	25.0	86.6	31.3	82.6	74.5
Decaussin-P., M. [88]	2017	187	16.6	17	14	135	21	40.0	88.8	45.2	86.5	79.7
Censi, S. [89]	2017	199	15.6	17	14	113	55	20.3	86.9	45.2	67.3	63.8
Macerola, E. [90]	2019	56	19.6	2	9	29	16	36.0	93.5	81.8	64.4	67.9
Wu, H. [91]	2019	41	17.1	4	3	25	9	25.0	86.2	68.3	42.9	73.5
**Total ^c^**		**1966**	**17.5**	**125**	**220**	**1261**	**360**	**37.9**	**91.0**	**63.8**	**77.8**	**75.3**

Definitive histology diagnosis: Ben, benign; Mal, malignant. N, number of samples; PPV, positive predictive value, NPV, negative predictive value. ^a^, DNA was extracted from tissue samples; ^b^, only RAS mutation positive nodules were considered. ^c^, Sum of data and diagnostic performance of sum of data.

**Table 3:** Distribution of RAS mutations in thyroid cancer of different differentiation level.

	Year	WDTC n/Total	WDTC %	PDTC n/Total	PDTC %	UTC n/Tot	UTC %
Lemoine, N.R. [24]	1989	8/15	53.3	6/10	60.0	-	-
Suarez, H.G. [22]	1988	8/13	61.5	1/10	10.0	-	-
Du Villard, J.A. [102]	1995	1/6	16.7	6/14	42.8	1/1	100
Bouras, M. [34]	1998	6/66	9.0	-	-	0/8	0.0
Ricarte-Filho, J.C. [103]	2009	1/11	9.0	8/55	14.5	0/2	0.0
Liu, Z. [104]	2008	13/64	20.3	-	-	4/51	7.8
Santarpia, L. [105]	2008	0/8	0.0	-	-	2/15	13.3
Manenti, G. [36]	1994	5/52	9.6	3/11	27.3	1/5	20.0
Garcia-Rostan, G. [45]	2003	4/49	8.2	16/29	55.2	15/29	51.7
Hou, P. [106]	2008	22/172	12.8	-	-	4/50	8.0
Basolo, F. [101]	2000	5/36	13.9	8/44	18.2	3/8	37.5
**Total**		**73/492**	**14.8**	**48/173**	**27.7**	**30/169**	**17.7**

## References

[1] Parsa A.A., Gharib H., Gharib H. (2018). Epidemiology of Thyroid Nodules. Thyroid Nodules. Contemporary Endocrinology.

[2] Perros P., British Thyroid Association, Royal College of Physicians (2007). Fine needle aspiration cytology (FNAC). Guidelines for the Management of Thyroid Cancer.

[3] Cibas E.S., Ali S.Z. (2017). The 2017 Bethesda System for Reporting Thyroid Cytopathology. Thyroid.

[4] Bongiovanni M., Spitale A., Faquin W.C., Mazzucchelli L., Baloch Z.W. (2012). The Bethesda System for Reporting Thyroid Cytopathology: A meta-analysis. Acta Cytol..

[5] Haugen B.R., Alexander E.K., Bible K.C., Doherty G.M., Mandel S.J., Nikiforov Y.E., Pacini F., Randolph G.W., Sawka A.M., Schlumberger M. (2015). 2015 American Thyroid Association Management Guidelines for Adult Patients with Thyroid Nodules and Differentiated Thyroid Cancer: The American Thyroid Association Guidelines Task Force on Thyroid Nodules and Differentiated Thyroid Cancer. Thyroid.

[6] Gharib H., Papini E., Garber J.R., Duick D.S., Harrell R.M., Hegedus L., Paschke R., Valcavi R., Vitti P. (2016). American Association of Clinical Endocrinologists, American College of Endocrinology, and Associazione Medici Endocrinologi Medical Guidelines for Clinical Practice for the Diagnosis and Management of Thyroid Nodules—2016 Update. Endocr. Pr..

[7] Nikiforov Y.E., Ohori N.P., Hodak S.P., Carty S.E., LeBeau S.O., Ferris R.L., Yip L., Seethala R.R., Tublin M.E., Stang M.T. (2011). Impact of mutational testing on the diagnosis and management of patients with cytologically indeterminate thyroid nodules: A prospective analysis of 1056 FNA samples. J. Clin. Endocrinol. Metab..

[8] Alexander E.K., Kennedy G.C., Baloch Z.W., Cibas E.S., Chudova D., Diggans J., Friedman L., Kloos R.T., LiVolsi V.A., Mandel S.J. (2010). Preoperative diagnosis of benign thyroid nodules with indeterminate cytology. N. Engl. J. Med..

[9] Beaudenon-Huibregtse S., Alexander E.K., Guttler R.B., Hershman J.M., Babu V., Blevins T.C., Moore P., Andruss B., Labourier E. (2014). Centralized molecular testing for oncogenic gene mutations complements the local cytopathologic diagnosis of thyroid nodules. Thyroid.

[10] Hall A. (1990). The cellular functions of small GTP-binding proteins. Science.

[11] Exton J.H. (1998). Small GTPases minireview series. J. Biol. Chem..

[12] Santelli G., de Franciscis V., Portella G., Chiappetta G., D’Alessio A., Califano D., Rosati R., Mineo A., Monaco C., Manzo G. (1993). Production of transgenic mice expressing the Ki-*ras* oncogene under the control of a thyroglobulin promoter. Cancer Res..

[13] Rochefort P., Caillou B., Michiels F.M., Ledent C., Talbot M., Schlumberger M., Lavelle F., Monier R., Feunteun J. (1996). Thyroid pathologies in transgenic mice expressing a human activated Ras gene driven by a thyroglobulin promoter. Oncogene.

[14] Garcia-Rendueles M.E., Ricarte-Filho J.C., Untch B.R., Landa I., Knauf J.A., Voza F., Smith V.E., Ganly I., Taylor B.S., Persaud Y. (2015). NF2 Loss Promotes Oncogenic *RAS*-Induced Thyroid Cancers via YAP-Dependent Transactivation of *RAS* Proteins and Sensitizes them to MEK Inhibition. Cancer Discov..

[15] Krishnamoorthy G.P., Davidson N.R., Leach S.D., Zhao Z., Lowe S.W., Lee G., Landa I., Nagarajah J., Saqcena M., Singh K. (2019). EIF1AX and *RAS* Mutations Cooperate to Drive Thyroid Tumorigenesis through ATF4 and c-MYC. Cancer Discov..

[16] Karunamurthy A., Panebianco F., Hsiao S.J., Vorhauer J., Nikiforova M.N., Chiosea S., Nikiforov Y.E. (2016). Prevalence and phenotypic correlations of EIF1AX mutations in thyroid nodules. Endocr. Relat. Cancer.

[17] Vitagliano D., Portella G., Troncone G., Francione A., Rossi C., Bruno A., Giorgini A., Coluzzi S., Nappi T.C., Rothstein J.L. (2006). Thyroid targeting of the N-*ras*(Gln61Lys) oncogene in transgenic mice results in follicular tumors that progress to poorly differentiated carcinomas. Oncogene.

[18] Guerra C., Mijimolle N., Dhawahir A., Dubus P., Barradas M., Serrano M., Campuzano V., Barbacid M. (2003). Tumor induction by an endogenous K-*ras* oncogene is highly dependent on cellular context. Cancer Cell.

[19] Ray K.C., Bell K.M., Yan J., Gu G., Chung C.H., Washington M.K., Means A.L. (2011). Epithelial tissues have varying degrees of susceptibility to K*ras*(G12D)-initiated tumorigenesis in a mouse model. PLoS ONE.

[20] Zhang J., Kong G., Rajagopalan A., Lu L., Song J., Hussaini M., Zhang X., Ranheim E.A., Liu Y., Wang J. (2017). p53-/- synergizes with enhanced N*ras*G12D signaling to transform megakaryocyte-erythroid progenitors in acute myeloid leukemia. Blood.

[21] O’Dell M.R., Huang J.L., Whitney-Miller C.L., Deshpande V., Rothberg P., Grose V., Rossi R.M., Zhu A.X., Land H., Bardeesy N. (2012). K*ras*(G12D) and p53 mutation cause primary intrahepatic cholangiocarcinoma. Cancer Res..

[22] Suarez H.G., Du Villard J.A., Caillou B., Schlumberger M., Tubiana M., Parmentier C., Monier R. (1988). Detection of activated *ras* oncogenes in human thyroid carcinomas. Oncogene.

[23] Lemoine N.R., Mayall E.S., Wyllie F.S., Farr C.J., Hughes D., Padua R.A., Thurston V., Williams E.D., Wynford-Thomas D. (1988). Activated *ras* oncogenes in human thyroid cancers. Cancer Res..

[24] Lemoine N.R., Mayall E.S., Wyllie F.S., Williams E.D., Goyns M., Stringer B., Wynford-Thomas D. (1989). High frequency of *ras* oncogene activation in all stages of human thyroid tumorigenesis. Oncogene.

[25] Ezzat S., Zheng L., Kolenda J., Safarian A., Freeman J.L., Asa S.L. (1996). Prevalence of activating *ras* mutations in morphologically characterized thyroid nodules. Thyroid.

[26] Sugg S.L., Ezzat S., Zheng L., Freeman J.L., Rosen I.B., Asa S.L. (1999). Oncogene profile of papillary thyroid carcinoma. Surgery.

[27] Soares P., Trovisco V., Rocha A.S., Lima J., Castro P., Preto A., Maximo V., Botelho T., Seruca R., Sobrinho-Simoes M. (2003). BRAF mutations and RET/PTC rearrangements are alternative events in the etiopathogenesis of PTC. Oncogene.

[28] Yoshimoto K., Iwahana H., Fukuda A., Sano T., Katsuragi K., Kinoshita M., Saito S., Itakura M. (1992). *Ras* mutations in endocrine tumors: Mutation detection by polymerase chain reaction-single strand conformation polymorphism. Jpn. J. Cancer Res..

[29] Naito H., Pairojkul C., Kitahori Y., Yane K., Miyahara H., Konishi N., Matsunaga T., Hiasa Y. (1998). Different *ras* gene mutational frequencies in thyroid papillary carcinomas in Japan and Thailand. Cancer Lett..

[30] Gabel H., Rehnqvist N. (1997). The Swedish National Donor Register. Transpl. Proc..

[31] Esapa C.T., Johnson S.J., Kendall-Taylor P., Lennard T.W., Harris P.E. (1999). Prevalence of Ras mutations in thyroid neoplasia. Clin. Endocrinol..

[32] Motoi N., Sakamoto A., Yamochi T., Horiuchi H., Motoi T., Machinami R. (2000). Role of *ras* mutation in the progression of thyroid carcinoma of follicular epithelial origin. Pathol. Res. Pr..

[33] Vasko V., Ferrand M., Di Cristofaro J., Carayon P., Henry J.F., de Micco C. (2003). Specific pattern of *RAS* oncogene mutations in follicular thyroid tumors. J. Clin. Endocrinol. Metab..

[34] Bouras M., Bertholon J., Dutrieux-Berger N., Parvaz P., Paulin C., Revol A. (1998). Variability of Ha-*ras* (codon 12) proto-oncogene mutations in diverse thyroid cancers. Eur. J. Endocrinol..

[35] Liu R.T., Hou C.Y., You H.L., Huang C.C., Hock L., Chou F.F., Wang P.W., Cheng J.T. (2004). Selective occurrence of *ras* mutations in benign and malignant thyroid follicular neoplasms in Taiwan. Thyroid.

[36] Manenti G., Pilotti S., Re F.C., Della Porta G., Pierotti M.A. (1994). Selective activation of *ras* oncogenes in follicular and undifferentiated thyroid carcinomas. Eur. J. Cancer.

[37] Giordano T.J., Beaudenon-Huibregtse S., Shinde R., Langfield L., Vinco M., Laosinchai-Wolf W., Labourier E. (2014). Molecular testing for oncogenic gene mutations in thyroid lesions: A case-control validation study in 413 postsurgical specimens. Hum. Pathol..

[38] Park J.Y., Kim W.Y., Hwang T.S., Lee S.S., Kim H., Han H.S., Lim S.D., Kim W.S., Yoo Y.B., Park K.S. (2013). *BRAF* and *RAS* mutations in follicular variants of papillary thyroid carcinoma. Endocr. Pathol..

[39] McFadden D.G., Dias-Santagata D., Sadow P.M., Lynch K.D., Lubitz C., Donovan S.E., Zheng Z., Le L., Iafrate A.J., Daniels G.H. (2014). Identification of oncogenic mutations and gene fusions in the follicular variant of papillary thyroid carcinoma. J. Clin. Endocrinol. Metab..

[40] Schulten H.J., Salama S., Al-Ahmadi A., Al-Mansouri Z., Mirza Z., Al-Ghamdi K., Al-Hamour O.A., Huwait E., Gari M., Al-Qahtani M.H. (2013). Comprehensive survey of *HRAS*, *KRAS*, and *NRAS* mutations in proliferative thyroid lesions from an ethnically diverse population. Anticancer Res..

[41] Jeong S.H., Hong H.S., Lee E.H., Kwak J.J., Lee J.Y. (2018). Analysis of *RAS* mutation in thyroid nodular hyperplasia and follicular neoplasm in a Korean population. Endocrinol. Diabetes Metab..

[42] Namba H., Rubin S.A., Fagin J.A. (1990). Point mutations of *ras* oncogenes are an early event in thyroid tumorigenesis. Mol. Endocrinol..

[43] Shi Y.F., Zou M.J., Schmidt H., Juhasz F., Stensky V., Robb D., Farid N.R. (1991). High rates of *ras* codon 61 mutation in thyroid tumors in an iodide-deficient area. Cancer Res..

[44] Nikiforova M.N., Lynch R.A., Biddinger P.W., Alexander E.K., Dorn G.W., Tallini G., Kroll T.G., Nikiforov Y.E. (2003). *RAS* point mutations and PAX8-PPAR gamma rearrangement in thyroid tumors: Evidence for distinct molecular pathways in thyroid follicular carcinoma. J. Clin. Endocrinol. Metab..

[45] Garcia-Rostan G., Zhao H., Camp R.L., Pollan M., Herrero A., Pardo J., Wu R., Carcangiu M.L., Costa J., Tallini G. (2003). *ras* mutations are associated with aggressive tumor phenotypes and poor prognosis in thyroid cancer. J. Clin. Oncol..

[46] Zhu Z., Gandhi M., Nikiforova M.N., Fischer A.H., Nikiforov Y.E. (2003). Molecular profile and clinical-pathologic features of the follicular variant of papillary thyroid carcinoma. An unusually high prevalence of *ras* mutations. Am. J. Clin. Pathol..

[47] Hara H., Fulton N., Yashiro T., Ito K., DeGroot L.J., Kaplan E.L. (1994). N-*ras* mutation: An independent prognostic factor for aggressiveness of papillary thyroid carcinoma. Surgery.

[48] Duan H., Liu X., Ren X., Zhang H., Wu H., Liang Z. (2019). Mutation profiles of follicular thyroid tumors by targeted sequencing. Diagn. Pathol..

[49] Schlumberger M.J. (1998). Papillary and follicular thyroid carcinoma. N. Engl. J. Med..

[50] Adeniran A.J., Zhu Z., Gandhi M., Steward D.L., Fidler J.P., Giordano T.J., Biddinger P.W., Nikiforov Y.E. (2006). Correlation between genetic alterations and microscopic features, clinical manifestations, and prognostic characteristics of thyroid papillary carcinomas. Am. J. Surg. Pathol..

[51] Gupta S., Ajise O., Dultz L., Wang B., Nonaka D., Ogilvie J., Heller K.S., Patel K.N. (2012). Follicular variant of papillary thyroid cancer: Encapsulated, nonencapsulated, and diffuse: Distinct biologic and clinical entities. Arch. Otolaryngol. Head Neck Surg..

[52] Zhao L., Dias-Santagata D., Sadow P.M., Faquin W.C. (2017). Cytological, molecular, and clinical features of noninvasive follicular thyroid neoplasm with papillary-like nuclear features versus invasive forms of follicular variant of papillary thyroid carcinoma. Cancer Cytopathol..

[53] Nikiforov Y.E., Seethala R.R., Tallini G., Baloch Z.W., Basolo F., Thompson L.D., Barletta J.A., Wenig B.M., Al Ghuzlan A., Kakudo K. (2016). Nomenclature Revision for Encapsulated Follicular Variant of Papillary Thyroid Carcinoma: A Paradigm Shift to Reduce Overtreatment of Indolent Tumors. JAMA Oncol..

[54] Bongiovanni M., Faquin W., Giovanella L., Durante C., Kopp P., Trimboli P. (2019). Impact of non-invasive follicular thyroid neoplasms with papillary-like nuclear features (NIFTP) on risk of malignancy in patients undergoing lobectomy/thyroidectomy for suspicious for malignancy or malignant fine-needle aspiration cytology findings: A systematic review and meta-analysis. Eur. J. Endocrinol..

[55] Ruanpeng D., Cheungpasitporn W., Thongprayoon C., Hennessey J.V., Shrestha R.T. (2019). Systematic Review and Meta-analysis of the Impact of Noninvasive Follicular Thyroid Neoplasm with Papillary-Like Nuclear Features (NIFTP) on Cytological Diagnosis and Thyroid Cancer Prevalence. Endocr. Pathol..

[56] Osamura R.Y., Klöppel G., Rosai J. (2017). WHO Classification of Tumours of Endocrine Organs.

[57] Howitt B.E., Chang S., Eszlinger M., Paschke R., Drage M.G., Krane J.F., Barletta J.A. (2015). Fine-needle aspiration diagnoses of noninvasive follicular variant of papillary thyroid carcinoma. Am. J. Clin. Pathol.

[58] Jiang X.S., Harrison G.P., Datto M.B. (2016). Young Investigator Challenge: Molecular testing in noninvasive follicular thyroid neoplasm with papillary-like nuclear features. Cancer Cytopathol..

[59] Cho U., Mete O., Kim M.H., Bae J.S., Jung C.K. (2017). Molecular correlates and rate of lymph node metastasis of non-invasive follicular thyroid neoplasm with papillary-like nuclear features and invasive follicular variant papillary thyroid carcinoma: The impact of rigid criteria to distinguish non-invasive follicular thyroid neoplasm with papillary-like nuclear features. Mod. Pathol..

[60] Proietti A., Sartori C., Macerola E., Borrelli N., Materazzi G., Vitti P., Basolo F. (2017). Low frequency of TERT promoter mutations in a series of well-differentiated follicular-patterned thyroid neoplasms. Virchows Arch..

[61] Borrelli N., Denaro M., Ugolini C., Poma A.M., Miccoli M., Vitti P., Miccoli P., Basolo F. (2017). miRNA expression profiling of ‘noninvasive follicular thyroid neoplasms with papillary-like nuclear features’ compared with adenomas and infiltrative follicular variants of papillary thyroid carcinomas. Mod. Pathol..

[62] Lee S.E., Hwang T.S., Choi Y.L., Kim W.Y., Han H.S., Lim S.D., Kim W.S., Yoo Y.B., Kim S.K. (2017). Molecular Profiling of Papillary Thyroid Carcinoma in Korea with a High Prevalence of BRAF(V600E) Mutation. Thyroid.

[63] Giannini R., Ugolini C., Poma A.M., Urpi M., Niccoli C., Elisei R., Chiarugi M., Vitti P., Miccoli P., Basolo F. (2017). Identification of Two Distinct Molecular Subtypes of Non-Invasive Follicular Neoplasm with Papillary-Like Nuclear Features by Digital RNA Counting. Thyroid.

[64] Ohori N.P., Wolfe J., Carty S.E., Yip L., LeBeau S.O., Berg A.N., Schoedel K.E., Nikiforov Y.E., Seethala R.R. (2017). The influence of the noninvasive follicular thyroid neoplasm with papillary-like nuclear features (NIFTP) resection diagnosis on the false-positive thyroid cytology rate relates to quality assurance thresholds and the application of NIFTP criteria. Cancer Cytopathol..

[65] Valderrabano P., Khazai L., Leon M.E., Thompson Z.J., Ma Z., Chung C.H., Hallanger-Johnson J.E., Otto K.J., Rogers K.D., Centeno B.A. (2017). Evaluation of ThyroSeq v2 performance in thyroid nodules with indeterminate cytology. Endocr. Relat. Cancer.

[66] Brandler T.C., Liu C.Z., Cho M., Zhou F., Cangiarella J., Yee-Chang M., Shi Y., Simsir A., Sun W. (2018). Does Noninvasive Follicular Thyroid Neoplasm With Papillary-Like Nuclear Features (NIFTP) Have a Unique Molecular Profile?. Am. J. Clin. Pathol..

[67] Song Y.S., Won J.K., Yoo S.K., Jung K.C., Kim M.J., Kim S.J., Cho S.W., Lee K.E., Yi K.H., Seo J.S. (2018). Comprehensive Transcriptomic and Genomic Profiling of Subtypes of Follicular Variant of Papillary Thyroid Carcinoma. Thyroid.

[68] Johnson D.N., Furtado L.V., Long B.C., Zhen C.J., Wurst M., Mujacic I., Kadri S., Segal J.P., Antic T., Cipriani N.A. (2018). Noninvasive Follicular Thyroid Neoplasms With Papillary-like Nuclear Features Are Genetically and Biologically Similar to Adenomatous Nodules and Distinct From Papillary Thyroid Carcinomas With Extensive Follicular Growth. Arch. Pathol. Lab. Med..

[69] Jung C.K., Kim Y., Jeon S., Jo K., Lee S., Bae J.S. (2018). Clinical utility of EZH1 mutations in the diagnosis of follicular-patterned thyroid tumors. Hum. Pathol..

[70] Kim T.H., Lee M., Kwon A.Y., Choe J.H., Kim J.H., Kim J.S., Hahn S.Y., Shin J.H., Chung M.K., Son Y.I. (2018). Molecular genotyping of the non-invasive encapsulated follicular variant of papillary thyroid carcinoma. Histopathology.

[71] Sohn S.Y., Lee J.J., Lee J.H. (2020). Molecular Profile and Clinicopathologic Features of Follicular Variant Papillary Thyroid Carcinoma. Pathol. Oncol. Res..

[72] Reinke R.H., Larsen S.R., Mathiesen J.S., Godballe C., Londero S.C. (2020). Noninvasive Follicular Thyroid Neoplasm with Papillary-Like Nuclear Features is Rare: A Population Based Study of Incidence. Head Neck Pathol..

[73] Cibas E.S., Ali S.Z. (2009). The Bethesda System for Reporting Thyroid Cytopathology. Thyroid.

[74] Alshaikh S., Harb Z., Aljufairi E., Almahari S.A. (2018). Classification of thyroid fine-needle aspiration cytology into Bethesda categories: An institutional experience and review of the literature. Cytojournal.

[75] Brown C., Mangano W., Thompson S., Richmond B. (2018). Factors Predicting Thyroid Malignancy in Fine Needle Aspiration Biopsy Specimens Classified as Atypia of Uncertain Significance/Follicular Lesion of Uncertain Significance. Am. Surg..

[76] Mondal S.K., Sinha S., Basak B., Roy D.N., Sinha S.K. (2013). The Bethesda system for reporting thyroid fine needle aspirates: A cytologic study with histologic follow-up. J. Cytol..

[77] Liu S., Gao A., Zhang B., Zhang Z., Zhao Y., Chen P., Ji M., Hou P., Shi B. (2014). Assessment of molecular testing in fine-needle aspiration biopsy samples: An experience in a Chinese population. Exp. Mol. Pathol..

[78] Rossi M., Buratto M., Tagliati F., Rossi R., Lupo S., Trasforini G., Lanza G., Franceschetti P., Bruni S., Degli Uberti E. (2015). Relevance of *BRAF*(V600E) Mutation Testing Versus *RAS* Point Mutations and RET/PTC Rearrangements Evaluation in the Diagnosis of Thyroid Cancer. Thyroid.

[79] Gill M.S., Nayan S., Kocovski L., Cutz J.C., Archibald S.D., Jackson B.S., Young J.E., Gupta M.K. (2014). Local molecular analysis of indeterminate thyroid nodules. J. Otolaryngol. Head Neck Surg..

[80] Eszlinger M., Bohme K., Ullmann M., Gorke F., Siebolts U., Neumann A., Franzius C., Adam S., Molwitz T., Landvogt C. (2017). Evaluation of a Two-Year Routine Application of Molecular Testing of Thyroid Fine-Needle Aspirations Using a Seven-Gene Panel in a Primary Referral Setting in Germany. Thyroid.

[81] Cantara S., Capezzone M., Marchisotta S., Capuano S., Busonero G., Toti P., Di Santo A., Caruso G., Carli A.F., Brilli L. (2010). Impact of proto-oncogene mutation detection in cytological specimens from thyroid nodules improves the diagnostic accuracy of cytology. J. Clin. Endocrinol. Metab..

[82] Moses W., Weng J., Sansano I., Peng M., Khanafshar E., Ljung B.M., Duh Q.Y., Clark O.H., Kebebew E. (2010). Molecular testing for somatic mutations improves the accuracy of thyroid fine-needle aspiration biopsy. World J. Surg..

[83] Stence A.A., Gailey M.P., Robinson R.A., Jensen C.S., Ma D. (2015). Simultaneously Detection of 50 Mutations at 20 Sites in the BRAF and *RAS* Genes by Multiplexed Single-Nucleotide Primer Extension Assay Using Fine-Needle Aspirates of Thyroid Nodules. Yale J. Biol. Med..

[84] De Napoli L., Bakkar S., Ambrosini C.E., Materazzi G., Proietti A., Macerola E., Basolo F., Miccoli P. (2016). Indeterminate Single Thyroid Nodule: Synergistic Impact of Mutational Markers and Sonographic Features in Triaging Patients to Appropriate Surgery. Thyroid.

[85] Shrestha R.T., Evasovich M.R., Amin K., Radulescu A., Sanghvi T.S., Nelson A.C., Shahi M., Burmeister L.A. (2016). Correlation Between Histological Diagnosis and Mutational Panel Testing of Thyroid Nodules: A Two-Year Institutional Experience. Thyroid.

[86] Eszlinger M., Krogdahl A., Munz S., Rehfeld C., Precht Jensen E.M., Ferraz C., Bosenberg E., Drieschner N., Scholz M., Hegedus L. (2014). Impact of molecular screening for point mutations and rearrangements in routine air-dried fine-needle aspiration samples of thyroid nodules. Thyroid.

[87] Patel S.G., Carty S.E., McCoy K.L., Ohori N.P., LeBeau S.O., Seethala R.R., Nikiforova M.N., Nikiforov Y.E., Yip L. (2017). Preoperative detection of *RAS* mutation may guide extent of thyroidectomy. Surgery.

[88] Decaussin-Petrucci M., Descotes F., Depaepe L., Lapras V., Denier M.L., Borson-Chazot F., Lifante J.C., Lopez J. (2017). Molecular testing of *BRAF*, *RAS* and *TERT* on thyroid FNAs with indeterminate cytology improves diagnostic accuracy. Cytopathology.

[89] Censi S., Cavedon E., Bertazza L., Galuppini F., Watutantrige-Fernando S., De Lazzari P., Nacamulli D., Pennelli G., Fassina A., Iacobone M. (2017). Frequency and Significance of Ras, Tert Promoter, and Braf Mutations in Cytologically Indeterminate Thyroid Nodules: A Monocentric Case Series at a Tertiary-Level Endocrinology Unit. Front. Endocrinol..

[90] Macerola E., Rago T., Proietti A., Basolo F., Vitti P. (2019). The mutational analysis in the diagnostic work-up of thyroid nodules: The real impact in a center with large experience in thyroid cytopathology. J. Endocrinol. Investig..

[91] Wu H., Zhang B., Cai G., Li J., Gu X. (2019). American College of Radiology thyroid imaging report and data system combined with K-*RAS* mutation improves the management of cytologically indeterminate thyroid nodules. PLoS ONE.

[92] Gupta N., Dasyam A.K., Carty S.E., Nikiforova M.N., Ohori N.P., Armstrong M., Yip L., LeBeau S.O., McCoy K.L., Coyne C. (2013). *RAS* mutations in thyroid FNA specimens are highly predictive of predominantly low-risk follicular-pattern cancers. J. Clin. Endocrinol. Metab..

[93] Nikiforov Y.E., Carty S.E., Chiosea S.I., Coyne C., Duvvuri U., Ferris R.L., Gooding W.E., Hodak S.P., LeBeau S.O., Ohori N.P. (2014). Highly accurate diagnosis of cancer in thyroid nodules with follicular neoplasm/suspicious for a follicular neoplasm cytology by ThyroSeq v2 next-generation sequencing assay. Cancer.

[94] Saavedra H.I., Knauf J.A., Shirokawa J.M., Wang J., Ouyang B., Elisei R., Stambrook P.J., Fagin J.A. (2000). The *RAS* oncogene induces genomic instability in thyroid PCCL3 cells via the MAPK pathway. Oncogene.

[95] Puzziello A., Guerra A., Murino A., Izzo G., Carrano M., Angrisani E., Zeppa P., Marotta V., Faggiano A., Vitale M. (2015). Benign thyroid nodules with *RAS* mutation grow faster. Clin. Endocrinol..

[96] Medici M., Kwong N., Angell T.E., Marqusee E., Kim M.I., Frates M.C., Benson C.B., Cibas E.S., Barletta J.A., Krane J.F. (2015). The variable phenotype and low-risk nature of *RAS*-positive thyroid nodules. BMC Med..

[97] Alexander E.K., Hurwitz S., Heering J.P., Benson C.B., Frates M.C., Doubilet P.M., Cibas E.S., Larsen P.R., Marqusee E. (2003). Natural history of benign solid and cystic thyroid nodules. Ann. Intern. Med..

[98] Kuma K., Matsuzuka F., Yokozawa T., Miyauchi A., Sugawara M. (1994). Fate of untreated benign thyroid nodules: Results of long-term follow-up. World J. Surg..

[99] Durante C., Costante G., Lucisano G., Bruno R., Meringolo D., Paciaroni A., Puxeddu E., Torlontano M., Tumino S., Attard M. (2015). The natural history of benign thyroid nodules. JAMA.

[100] Guerra A., Di Crescenzo V., Garzi A., Cinelli M., Carlomagno C., Tonacchera M., Zeppa P., Vitale M. (2013). Genetic mutations in the treatment of anaplastic thyroid cancer: A systematic review. BMC Surg..

[101] Basolo F., Pisaturo F., Pollina L.E., Fontanini G., Elisei R., Molinaro E., Iacconi P., Miccoli P., Pacini F. (2000). N-*ras* mutation in poorly differentiated thyroid carcinomas: Correlation with bone metastases and inverse correlation to thyroglobulin expression. Thyroid.

[102] Du Villard J.A., Schlumberger M., Wicker R., Caillou B., Rochefort P., Feunteun J., Monier R., Parmentier C., Suarez H.G. (1995). Role of *ras* and gsp oncogenes in human epithelial thyroid tumorigenesis. J. Endocrinol. Investig..

[103] Ricarte-Filho J.C., Ryder M., Chitale D.A., Rivera M., Heguy A., Ladanyi M., Janakiraman M., Solit D., Knauf J.A., Tuttle R.M. (2009). Mutational profile of advanced primary and metastatic radioactive iodine-refractory thyroid cancers reveals distinct pathogenetic roles for BRAF, PIK3CA, and AKT1. Cancer Res..

[104] Liu Z., Hou P., Ji M., Guan H., Studeman K., Jensen K., Vasko V., El-Naggar A.K., Xing M. (2008). Highly prevalent genetic alterations in receptor tyrosine kinases and phosphatidylinositol 3-kinase/akt and mitogen-activated protein kinase pathways in anaplastic and follicular thyroid cancers. J. Clin. Endocrinol. Metab..

[105] Santarpia L., El-Naggar A.K., Cote G.J., Myers J.N., Sherman S.I. (2008). Phosphatidylinositol 3-kinase/akt and *ras*/raf-mitogen-activated protein kinase pathway mutations in anaplastic thyroid cancer. J. Clin. Endocrinol. Metab..

[106] Hou P., Ji M., Xing M. (2008). Association of PTEN gene methylation with genetic alterations in the phosphatidylinositol 3-kinase/AKT signaling pathway in thyroid tumors. Cancer.

[107] Fukahori M., Yoshida A., Hayashi H., Yoshihara M., Matsukuma S., Sakuma Y., Koizume S., Okamoto N., Kondo T., Masuda M. (2012). The associations between *RAS* mutations and clinical characteristics in follicular thyroid tumors: New insights from a single center and a large patient cohort. Thyroid.

[108] Jang E.K., Song D.E., Sim S.Y., Kwon H., Choi Y.M., Jeon M.J., Han J.M., Kim W.G., Kim T.Y., Shong Y.K. (2014). N*RAS* codon 61 mutation is associated with distant metastasis in patients with follicular thyroid carcinoma. Thyroid.

[109] Yip L., Nikiforova M.N., Yoo J.Y., McCoy K.L., Stang M.T., Armstrong M.J., Nicholson K.J., Ohori N.P., Coyne C., Hodak S.P. (2015). Tumor genotype determines phenotype and disease-related outcomes in thyroid cancer: A study of 1510 patients. Ann. Surg..

[110] Yoo S.K., Song Y.S., Lee E.K., Hwang J., Kim H.H., Jung G., Kim Y.A., Kim S.J., Cho S.W., Won J.K. (2019). Integrative analysis of genomic and transcriptomic characteristics associated with progression of aggressive thyroid cancer. Nat. Commun..

[111] Song Y.S., Lim J.A., Choi H., Won J.K., Moon J.H., Cho S.W., Lee K.E., Park Y.J., Yi K.H., Park D.J. (2016). Prognostic effects of TERT promoter mutations are enhanced by coexistence with *BRAF* or *RAS* mutations and strengthen the risk prediction by the ATA or TNM staging system in differentiated thyroid cancer patients. Cancer.

[112] Liu R., Xing M. (2016). *TERT* promoter mutations in thyroid cancer. Endocr. Relat. Cancer.

[113] Yin D.T., Yu K., Lu R.Q., Li X., Xu J., Lei M., Li H., Wang Y., Liu Z. (2016). Clinicopathological significance of *TERT* promoter mutation in papillary thyroid carcinomas: A systematic review and meta-analysis. Clin. Endocrinol..

[114] The Cancer Genome Atlas Research Network (2014). Integrated genomic characterization of papillary thyroid carcinoma. Cell.

[115] Shen X., Liu R., Xing M. (2017). A six-genotype genetic prognostic model for papillary thyroid cancer. Endocr. Relat. Cancer.

